# The Coming Southern Medical Association

**Published:** 1915-05

**Authors:** Edward H. Cary

**Affiliations:** Dallas, Texas


### The Coming Southern Medical Association.

DR. EDWARD H. CARY, DALLAS, TEXAS.

The coming of the Southern Medical Association to Dallas the week following the next State Fair means a great deal to Texas. It will be the first time a medical association of national importance has visited Texas, with one exception—the National Railroad Surgeons' Convention, which met in Galveston some fifteen years ago.

The leading men of Baltimore, New Orleans, Atlanta, Louisville and such cities will be in Dallas, much to the enjoyment of the many Texans who have the honor of their acquaintance. The public meetings will be particularly attractive. Monday, Tuesday and Wednesday nights, from 8 to 10 o'clock, will be occupied by two speakers each evening, all of whom have a national reputation, discussing live topics of public and professional value. The general entertainment committee has planned a great many interesting social affairs which will not conflict with any of the scientific work.

It is to be remembered that the Texas State Medical Association extended this invitation to the Southern Medical Society to visit Texas and, incidentally, Dallas. It is hoped that the profession generally will remember that after all they are the hosts. It is understood that Dallas will need no help financially, but will certainly count upon the profession generally to be upon the reception committee and make the visiting doctors feel at home and so pleased with the reception extended them that they may contribute their influence to bring the A. M. A. to Texas in 1917.

These great gatherings are helpful in many ways. First, the public is greatly benefited, and along with this the profession in and out of the State is mutually enlightened. It is understood that a promise was made that 1000 members would be the net result of the visit of the Southern Medical Society to Texas. To do this, something like 750 new members will have to be added.

